# Multi-Stage Data Processing for Enhancing Korean Cattle (Hanwoo) Weight Estimations by Automated Weighing Systems

**DOI:** 10.3390/ani15121785

**Published:** 2025-06-17

**Authors:** Dong-Hyeon Kim, Jae-Woo Song, Hyunjin Cho, Mingyung Lee, Dae-Hyun Lee, Seongwon Seo, Wang-Hee Lee

**Affiliations:** 1 Department of Smart Agriculture Systems, Chungnam National University, Daejeon 34134, Republic of Korea; dhyeonk99@gmail.com; 2 Department of Smart Agriculture Systems Machinery Engineering, Chungnam National University, Daejeon 34134, Republic of Korea; coega23@gmail.com (J.-W.S.); leedh7@cnu.ac.kr (D.-H.L.); 3 Division of Animal and Dairy Sciences, Chungnam National University, Daejeon 34134, Republic of Korea; chohyunjin0927@gmail.com (H.C.); mingyung1203@gmail.com (M.L.)

**Keywords:** cattle weight, data processing, Hanwoo, livestock automation, statistics

## Abstract

Weight is the most basic and important indicator in cattle management, and automation of its measurement serves as a fundamental step toward modern smart livestock farming. Automated weighing systems (AWS) capable of continuously measuring cattle weight, even during movement, have been explored as key monitoring components in smart livestock farming. However, owing to the high measurement variability caused by environmental factors, the accuracy of AWSs has been questioned. These factors include real-time fluctuations due to animal activities (e.g., feeding and locomotion), as well as measurement errors caused by residual feed or excreta within the AWS. Therefore, this study aimed to develop an algorithm to enhance the reliability of steer weight measurements using an AWS, ensuring close alignment with actual cattle body weight. Accordingly, daily weight data from 36 Hanwoo steers were processed using a three-stage approach consisting of outlier detection and removal, weight estimation, and post-processing for weight adjustment. The best-performing algorithm that combined Tukey’s fences for outlier detection, mean-based estimation, and post-processing based on daily weight gain recommended by the National Institute of Animal Science achieved a root mean square error of 12.35 kg, along with an error margin of less than 10% for individual steers. Overall, the study concluded that the AWS measured steer weight with high reliability through the developed algorithm, thereby contributing to data-driven intelligent precision feeding.

## Simple Summary

Automatic weight measurement is crucial in smart livestock management, as cattle weight serves as a key indicator of health and productivity. This study presents a robust three-stage data processing algorithm designed to enhance the accuracy of an automated weighing system (AWS) for real-time cattle weight tracking. The algorithm incorporates outlier removal followed by weight estimation and post-processing for weight correction to minimize measurement variability. The optimized algorithm achieved a root mean square error (RMSE) of 12.35 kg with an error margin below 10%, demonstrating that an AWS enhanced with this algorithm can reliably deliver precise weight measurements. This advancement enables more data-driven and intelligent livestock management decisions.

## 1. Introduction

In livestock farming, body weight is a key indicator in decision making. Farmers seek to optimize feeding and management strategies based on weight to enhance economic viability and operational efficiency [1]. By measuring and monitoring body weight, farmers can estimate the health status of livestock, evaluate product quality at the time of shipment, and leverage data for various purposes [2,3]. Traditionally, body weight is measured manually using a weighing scale at intervals of several months. However, this process is labor-intensive and causes stress in cattle owing to repeated measurements in a limited space.

To address these challenges, efforts have been made to introduce automated weighing systems (AWSs) that measure cattle weights in real time without high labor demands and stress on cattle [4,5]. One of the most representative AWS implementations is the walk-over weighing system, which has been validated for various livestock species such as cattle, sheep, and pigs [3,4,5,6]. In grazing systems, animals are weighed as they pass over the scale when entering or exiting through fences, whereas in housed systems, body weight is measured when animals step on or walk over the scale to perform routine behaviors such as feed intake. The system typically identifies individual animals by linking the scale to Radio-Frequency Identification (RFID) tags attached to each animal and automatically records their body weights. Recently, efforts have also been made to estimate body weight using image and video processing technologies [7,8]. These technologies have been increasingly demanded and implemented as part of smart livestock farming initiatives in numerous countries, including major cattle-producing nations such as the United States, Australia, and Brazil [4,9,10,11,12]. However, considering that an AWS measures body weight in a real-world feeding environment where animals move freely, raw data obtained from an AWS are not directly suitable for use in accurate body weight measurements because of significant variations and error sources, such as residual feed, excrement, and other factors [13]. Compared to the traditional static weighing scale, which captures a single-point measurement at a specific time, the AWS continuously collects large-scale time-series data to measure steer weight. The recorded weight fluctuates in real time owing to livestock activities, such as feeding and movement, and variations may also arise from residual feed or waste present within the AWS [4]. These fluctuations and outliers compromise the reliability of AWS-based weight measurements; thus, unprocessed data causes biased decision making in livestock management [14]. This highlights the need to improve the quality of livestock monitoring data and to implement strategies that enhance the reliability of AWS-based weight measurements by applying data processing methods [15].

Several studies have investigated methods for detecting anomalies in time-series data within the livestock and dairy sectors, such as monitoring daily milk production in dairy cows and identifying feeding outliers in cattle [16,17]. However, owing to the difficulty of obtaining automated weight measurements and the limited accessibility of relevant data, none of these studies applied these methods to improve the reliability of automated weight measurements in cattle [18,19]. Furthermore, owing to the harsh conditions of actual farming environments, user convenience is often compromised, thus indicating the need for simple algorithms that are readily applicable to individual farms, rather than complex algorithmic forms. Therefore, it is necessary to apply simple yet powerful statistical principles at each stage of data processing in order to increase measurement accuracy and develop a data processing protocol for the AWS that allows easy access for actual farm users. This study aimed to process real-time livestock weight data obtained from an AWS to derive a highly reliable measurement that closely approximates the actual body weight of Hanwoo steers. Outliers were identified using widely recognized statistical criteria, and weight estimates were obtained using multiple approaches, followed by practical post-data processing. This study will enhance the field applicability of the AWS and will subsequently contribute to the establishment of key technologies required for the implementation of smart farming and the advancement of livestock farming [20].

## 2. Materials and Methods

### 2.1. Data Acquisition

Data were collected from the Center for Animal Science Research, Chungnam National University (Daejeon, Republic of Korea). The use of animals and protocols for this experiment were reviewed and pre-approved by the Chungnam National University Animal Research Ethics Committee (CNU-01021). The static weighing scale measures individual weights under uniformly controlled conditions. The AWS (Dawoon Co., Incheon, Republic of Korea) measures the weight of cattle while they remain at or feed from an automated concentrate feeder station. This station is equipped with a load cell sensor that records both the time of entry and the duration of the animal’s stay. A total of 48,167 data points were obtained using the AWS, which measured the real-time body weight of 36 Hanwoo steers assigned with Radio-Frequency Identification. Measurements were collected continuously, 24 h a day, for one week in the middle of each month from February to April 2019. These data points served as reference values for the actual weight of the cattle and were recorded on the day that represented the midpoint of the week. The weight was measured monthly using an AWS.

### 2.2. Development of Algorithmic Process for Weight Estimation

An algorithmic process was developed to determine the body weight of cattle by identifying the AWS measurements with the least errors compared with the reference static weighing scale measurements. This algorithm consisted of three main steps: (1) outlier removal through data pre-processing, (2) weight estimation and comparison with reference measurements, and (3) weight correction through data post-processing.

### 2.3. Data Pre-Processing

During the initial screening, data collected from the forage intake monitoring system concurrently with body weight measurements were excluded. Thereafter, 11,657 weight records consisting of up to 28 measurements for each individual were retained. Outliers that could cause biased results were detected using two statistic-based techniques, which are relatively simple yet effective, after reviewing several methods [17]. Tukey’s fences method, which detects outliers based on the interquartile range (IQR), was employed. In this method, data smaller than the value obtained by subtracting 1.5 × the IQR from the first quartile or data greater than the value obtained by adding 1.5 × the IQR to the third quartile were removed [21]. As another option, data that were 1.5 times the standard deviation away from the mean were considered outliers to account for the probability range of a normal distribution and to prevent over- and under-detection [17]. A total of 1474 and 783 outliers were removed using the first and second outlier detection methods, respectively, with an average of approximately 11 data points per cow. Finally, the dataset was converted into daily averages for each individual to prevent bias due to data imbalances in subsequent processes.

### 2.4. Weight Estimation for Automatic Weighing System

The main purpose of weight estimation was to determine the representative value measured by the AWS. This value showed the least discrepancy from the reference weight measured using the static weight scale. The following three indices were employed for the pre-processed monthly AWS weight measurements: (1) average, (2) median, and (3) predictive value from the linear regression analysis (LinearR). The mean and median values for each seven-day period within each month were calculated and used as representative measurements for each cow. These representative measurements were compared with the reference static weight scale measurements that were taken at mid-date. To predict the weight for the mid-date (4th day), a regression analysis was conducted using the seven-day data, excluding the 4th day. Owing to the requirement of a minimum amount of data for regression analysis, the analysis was only applied to cases with four or more data points.

### 2.5. Data Post-Processing

To enhance the reliability of the analysis, the estimated weights were further adjusted through data post-processing. The process considered conditions related to the actual weight based on the cattle average daily gain [22]. Data post-processing was performed by establishing a practical threshold based on the target daily weight gain for Hanwoo cattle provided by the National Institute of Animal Science (NIAS, Wanju, Korea). To account for monthly variations and prevent overfiltering, the threshold was set by multiplying the daily weight gain by a factor of 30 (Equation (1)). Based on this threshold, data that exceeded or fell below the threshold were removed.

Threshold = ± max(ADGBW) × 30(1)

where ADGBW is the target daily weight gain provided by the NIAS.

### 2.6. Performance Evaluation

Performance was evaluated using the root mean square error (RMSE) of the reference static scale weight measurement and the number of estimations within a 5% and 10% margin of error from the reference value [23]. The RMSE was used to assess the accuracy of estimating the weight of individual steers using the AWS, whereas the margin was used to evaluate the overall measurability of the AWS-based weight estimation for the entire population.

### 2.7. Statistical Analysis

Statistical analyses were conducted to compare the steer weights measured using the different methods. Several statistical comparisons were performed to account for various scenarios. First, the weight distribution of the population was assumed to follow a normal distribution, and normality was satisfied according to the central limit theorem given a sufficient number of individuals and data points [24,25]. To assess the group differences, the homogeneity of variances was initially tested using F-statistics, and statistical comparisons were made after confirming the homogeneity assumption. Moreover, Dunnett’s test was conducted to compare whether the weight measurements of each method differed from the reference static scale weight measurements. Furthermore, Tukey’s test was used for multiple comparisons between methods. Additionally, to account for the effect of individual IDs and to minimize errors due to non-normality, a non-parametric approach was employed using the Kruskal–Wallis test and Dunn’s test. These combined methods ensured a comprehensive comparison that considered the key assumptions of the statistical analysis, including normality, homogeneity of variances, and data characteristics. Statistical significance was set at *p* < 0.05, and the R software package version 4.4.2 [26] was used for statistical analysis [27].

## 3. Results

### 3.1. Comparison of Discrepancies Between AWSs and Static Scale Steer Weight Measurements

When evaluating the discrepancy between AWS-based weight measurements and the reference weight, the RMSE ranged from a minimum of 12.34 kg to a maximum of 18.35 kg. The maximum RMSE was achieved via standard deviation-based data pre-processing and median-based estimation in February, whereas the minimum RMSE was achieved via standard deviation-based data pre-processing and mean-based estimation in April (Table 1).

**Figure 1:**
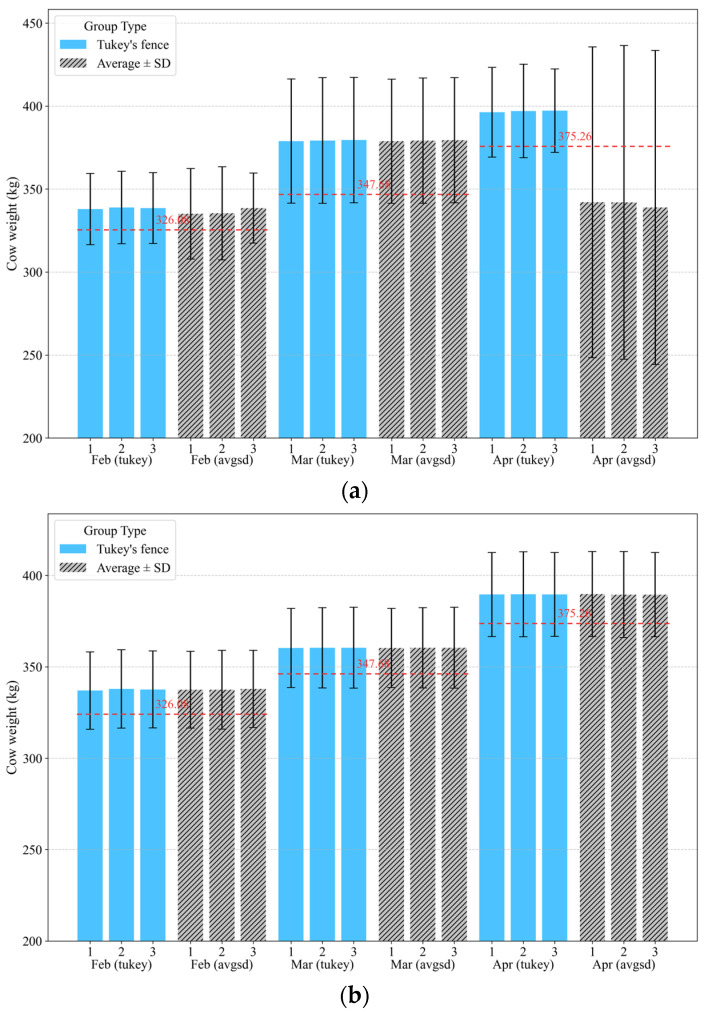
Comparison of three methods for estimating cow weight. (**a**) Before post-processing and (**b**) after post-processing. The reference weight is indicated by the red dashed line and its corresponding value, whereas the standard deviation for each weight is represented by the error bars.

The RMSE for the weight estimation methods was consistently the lowest in February but increased each month, reaching the highest value in April. The overall RMSE ranged from 14.54 kg to 15.08 kg depending on the pre-processing and estimation methods, with an average weight difference of 8.42 kg, which did not differ significantly between methods. Comparison of the results before and after post-processing revealed that post-processing enabled more consistent and accurate measurements. In particular, the variation in the April weight measurements and errors relative to the reference measurements were considerably larger without post-processing, thus highlighting the necessity for post-processing (Figure 1a). Nevertheless, regardless of post-processing, statistical analyses accounting for factors, such as individual variation (ID), normality, and methodological differences, revealed no significant differences between the three methods for estimating steer weight using the AWS and the reference weight (*p* > 0.05) (Figure 1b).

Depending on the pre-processing method, the approach using Tukey’s fences showed better predictive performance with mean- and regression-based methods, whereas the median-based estimation performed better with pre-processing using the mean and standard deviation. The mean-based estimation combined with Tukey’s fences consistently exhibited the lowest RMSE, whereas the weight predicted using regression after pre-processing with the mean and standard deviation exhibited the highest RMSE. Collectively, after applying post-processing, the standard deviation for all results decreased to within 30 kg, and the RMSE also decreased to below 20 kg. This demonstrates the methodological robustness for measuring steer weight using AWSs with post-processing.

### 3.2. Comparison of Individual Weight by Using Error Margin

**Figure 2:**
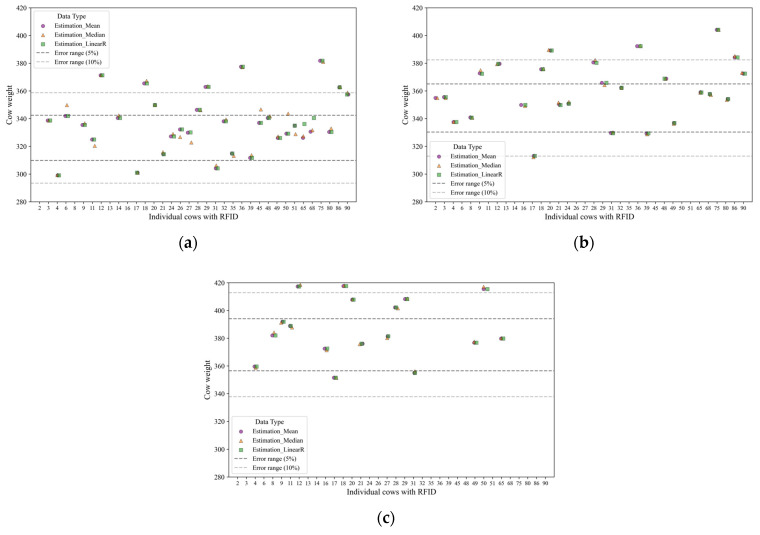
Individual weight estimation using statistical methods. (**a**) February, (**b**) March, and (**c**) April.

To analyze the accuracy of the individual measurements, each measured value was compared with the actual weight (individual reference weight), and the differences from the actual values were calculated (Figure 2).

Individually, the difference between the AWS and static scale steer weight measurements was 0.14 kg in the smallest case, whereas the largest difference was 35.94 kg. Overall, approximately 78% of the AWS weight measurements were within a 5% margin of error from the static-scale measurements, and all AWS-based measurements were within a 10% error range (Table 2).

When comparing the accuracy differences based on outlier detection methods prior to applying post-processing, the use of Tukey’s fence demonstrated better performance, with a difference of –0.6% to +28.2% compared with that of the standard deviation-based method. The average accuracy by weight estimation methods was confirmed to be 58.2–61.3% at an error range of ±5%, and 81.6–83.4% at an error range of ±10%. However, the median exhibited the lowest accuracy for both intervals. After post-processing, all methods were able to estimate individual weights within a 10% margin of error from the actual weight measured using the static scale. The statistical comparisons showed no significant differences from the reference values for each method, and the individual measurements showed a low error range. Therefore, the developed algorithm appears to be capable of processing AWS-measured values to produce results similar to the actual weight of the cattle. Notably, the combination of pre-processing using Tukey’s fence and mean-based weight estimation, followed by post-processing, proved to be the most robust approach, suggesting its promising potential as an optimal method for estimating steer weight using AWS.

## 4. Discussion

The weight of cattle serves as a critical assessment metric for the economic value of each individual and is widely utilized in informed management decision-making processes across most farms [28]. The AWS collects cattle body weight data and has lower labor requirements than those of conventional static weighing scales. The system enables continuous real-time monitoring and the generation of time-series data by tracking cattle during movement and feed intake, rather than providing a single-point measurement at a specific time. The body weight data obtained using this method serve as a fundamental metric for feed management in smart livestock systems [29].

Despite the substantial advantages of AWSs in advancing smart livestock farming and enhancing the efficiency of livestock management, AWS implementation poses challenges that must be addressed, particularly challenges concerning the reliability of weight measurements obtained automatically in dynamic environments in which cattle are in motion must be tackled [4]. Weight measurements obtained using AWSs frequently deviate from the actual body weight, with considerable variations owing to factors such as residual feed, feces, and the behavioral characteristics of cattle [4]. Accordingly, eliminating the sources of variation through constraints on the measurement environment is a prerequisite for the effective use of AWSs. However, as farm size increases, controlling factors, such as cattle physiology and behavior, during each measurement becomes inefficient and practically impossible. Therefore, given the numerous practical constraints in controlling the measurement environment, implementing a data processing algorithm that refines the measured data to closely match the actual value would enable the effective use of AWSs.

Data obtained from livestock facilities often exhibit substantial variability owing to the harsh measurement environment, consequently raising concerns about the reliability of the measurements. Such environmental factors include residual feed or manure remaining on the weighing platform, animal movement or posture during measurement, and the simultaneous presence of multiple animals on the scale, all of which can influence the recorded weight. Thus, the application of appropriate data pre-processing methods is essential for improving data quality [16]. Two pre-processing methods were used in this study. Although the differences in the measurements depending on the pre-processing method were not significant, the primary difference was their robustness against extreme values. In the dataset with the greatest deviation (April data), Tukey’s fence showed stronger robustness than that of the standard deviation-based data pre-processing method. As the standard deviation increased, the range of values considered normal expanded, thereby reducing the number of data points identified as outliers. Conversely, Tukey’s fence detected the highest number of outliers in April, which resulted in a 28.2% difference in prediction accuracy. However, excessive adjustment of the criteria to improve accuracy can lead to data loss. This emphasizes the importance of selecting an appropriate method based on the dataset characteristics. Before post-processing, the differences in the measurement values appeared to be influenced more by the number of data points (measured monthly) than by pre-processing or statistical measurement methods. This was confirmed by the results observed in March, which utilized the same data after pre-processing. Although pre-processing had already removed extreme values, the statistical methods seemed to have a minimal impact on the differences. In contrast, limited data points and monthly data variations appear to have a greater effect on weight prediction, thereby highlighting the importance of reducing data variability or noise before applying statistical methods.

Although post-processing is commonly used in fields such as image and frequency data analysis to facilitate result interpretation and visualization, its application to numerical datasets has rarely been reported [30,31]. In this study, postprocessing markedly improved the accuracy of individual steer weight estimation, thus ensuring that all measurements fell within a 10% error margin of the actual weight. Furthermore, post-processing substantially reduced the measurement variability, demonstrating a robust method for estimating steer weight that holds promising potential for AWS-based smart livestock farming applications. This suggests that post-processing can correct values that were not filtered during pre-processing. In scenarios of errors due to measurement conditions or device malfunctions, such as automated cattle weight estimation, post-processing can serve as a valuable tool. The effectiveness of post-processing in this study appears to be attributable to the use of criteria designed based on real-world standards specific to cattle species, rather than relying solely on data-driven methods [32]. This highlights the importance of adopting physiology- and biology-driven post-processing standards in environments, such as livestock farms, where the consideration of physiological and biological characteristics is crucial for accurate and reliable measurements [33]. According to the review by Qiao et al. [14], most previous studies presented RMSE values exceeding 20 kg, with some ranging between approximately 7.5 kg and 49.20 kg depending on the measurement perspective and sensor configuration. In this context, the RMSE of 12.35 kg achieved in our study serves as a reasonable indicator of accuracy and reinforces the credibility of the proposed approach.

Finally, it is worth noting that the dataset used in the analysis was relatively limited, with data collected over a three-week period and involving 36 animals matched with reference data. While this may restrict broader generalization, the sample size is comparable to those used in previous studies on Hanwoo and beef cattle under similar experimental conditions, where relatively small cohorts were also employed [14]. In such cases, including ours, the continuous collection of high-resolution feed intake data from each individual provided sufficient volume and granularity to support robust statistical analysis despite the limited number of animals. In addition, in light of the central limit theorem, which suggests that samples exceeding 30 observations typically approximate a normal distribution, the statistical methods applied in this study are deemed appropriate [25]. However, further validation using larger and more diverse datasets is warranted to reinforce the robustness and applicability of our findings.

## 5. Conclusions

Automated weight measurement is a critical component of smart livestock management. However, the accurate determination of cattle weight remains a challenge, often resulting in reliability issues with field-acquired data. To address this, this study introduces a novel algorithm designed to calibrate the AWS weight measurements, thereby enhancing their accuracy in approximating actual cattle weights. Although the dataset and number of cattle used in this study were limited, and the developed method may be applicable only to specific systems and breeds because of its post-processing criteria, the study presents an important algorithmic process that can serve as a foundational platform for automated weight measurement. By refining the data pre- and post-processing criteria and increasing the volume of data collected over an extended period for specific cattle breeds, this algorithm can be adapted to various AWS types. In addition, to ensure robust and generalizable application, the developed algorithm needs to be applied to data that account for variables related to changes in body weight, such as weight gain or loss in cattle. This adaptability, along with the proposed future work, underscore its practical applicability in enabling reliable automated weight measurement, which is a fundamental requirement for smart livestock farming.

## Figures and Tables

**Table 1:** Comparison of cow weight (kg) measurements using an automated weighing system and static scale.

Pre- Processing	Estimation Methods ^1^	Feb (228 ^2^)			Mar (190)		
		Predicted ^3^	Reference	RMSE ^4^	Predicted	Reference	RMSE
Tukey	Mean	337.01 ^a^	326.08 ^a^ (19.9)	12.35	360.31 ^c^	347.68 ^c^ (21.52)	14.39
		(21.14)			(21.68)		
	Median	337.92 ^a^		12.85	360.42 ^c^		14.64
		(21.44)			(21.98)		
	LinearR	337.64 ^a^		12.95	360.46 ^c^		14.44
		(21.01)			(22.11)		
Average ± standard deviation	Mean	337.48 ^b^	326.08 ^b^ (19.9)	13.01	360.31 ^d^	347.68 ^d^ (21.52)	14.39
		(20.95)			(21.68)		
	Median	337.49 ^b^		12.34	360.42 ^d^		14.64
		(21.51)			(21.98)		
	LinearR	337.91 ^b^		13.56	360.46 ^d^		14.44
		(21.15)			(22.11)		
		**Apr (144)**			**Total (562)**		
		**Predicted**	**Reference**	**RMSE**	**Predicted**	**Reference**	**RMSE**
Tukey	Mean	389.56 ^e^	375.26 ^e^ (19.48)	17.95	357.43 ^g^	349.28 ^g^ (28.53)	14.54
		(22.95)			(29.82)		
	Median	389.73 ^e^		18.23	357.89 ^g^		14.88
		(23.25)			(29.82)		
	LinearR	389.57 ^e^		17.95	357.71 ^g^		14.78
		(22.95)			(29.8)		
Average ± standard deviation	Mean	389.85 ^f^	375.26 ^f^ (19.48)	18.35	357.69 ^h^	349.28 ^h^ (28.53)	14.89
		(23.25)			(29.76)		
	Median	389.53 ^f^		18.09	357.66 ^h^		14.66
		(23.56)			(29.97)		
	LinearR	389.50 ^f^		18.20	357.81 ^h^		15.08
		(23)			(29.75)		

^1^ The same letters within a given method indicate that the methods are not significantly different within the same month at a significance level of 0.05, as determined by Dunnett’s test, Tukey’s test, and the Kruskal–Wallis test followed by Dunn’s post hoc analysis. ^2^ Number of data points used for estimating the automated weighing system after applying pre- and post-processing. ^3^ The predicted and reference values for each method are presented as averages with standard deviations in parentheses. ^4^ RMSE stands for root mean square error, whereas LinearR refers to linear regression analysis.

**Table 2:** Ratio (%) of AWS- and static scale-measured cow weights within the error margin.

Before post-processing (702 ^1^)							
Processing	Range	Feb			Mar		
		Mean	Median	LinearR	Mean	Median	LinearR
Tukey	5%	81.8	81.8	78.8	61.1	55.6	60
	10%	97	97	97	72.2	72.2	71.4
Avg ± SD	5%	73.5	82.4	72.7	61.1	55.6	60
	10%	94.1	94.1	97	72.2	72.2	71.4
Difference		8.3	−0.6	6.1	0	0	0
		2.9	2.9	0	0	0	0
		**Apr**			**Total**		
Tukey	5%	50	42.3	54.2	64.3	59.9	64.3
	10%	88.5	88.5	95.8	85.9	85.9	88.1
Avg ± SD	5%	40	31.4	41.2	58.2	56.5	58
	10%	65.7	65.7	67.6	77.3	77.3	78.7
Difference		10	10.9	13	6.1	3.4	6.3
		22.8	22.8	28.2	8.6	8.6	9.4
**After post-processing (562 ^1^)**							
**Processing**	**Range**	**Feb**			**Mar**		
		**Mean**	**Median**	**LinearR**	**Mean**	**Median**	**LinearR**
Tukey	5%	84.4	84.4	81.3	84.6	76.9	84
	10%	100	100	100	100	100	100
Avg ± SD	5%	78.1	87.5	75	84.6	76.9	84
	10%	100	100	100	100	100	100
Difference		6.3	−3.1	6.3	0	0	0
		0	0	0	0	0	0
		**Apr**			**Total**		
Tukey	5%	72.2	61.1	72.2	80.4	74.1	79.2
	10%	100	100	100	100	100	100
Avg ± SD	5%	77.8	61.1	77.8	80.2	75.2	78.9
	10%	100	100	100	100	100	100
Difference		−5.6	0	−5.6	0.2	−1.1	0.3
		0	0	0	0	0	0

^1^ Total number of data points used to estimate the automated weighing system.

## Data Availability

The original contributions presented in this study are included in the article. Further inquiries can be directed to the corresponding author(s).

## Abbreviations

The following abbreviations are used in this manuscript:

AWS	Automated weighing system
RMSE	Root mean square error
IQR	Interquartile range
LinearR	Linear regression
Avg ± SD	Average ± standard deviation
